# Supplementary material for: Chromothripsis during telomere crisis is independent of NHEJ, and consistent with a replicative origin
Source: Genome Res. 2019 May;29(5):737–49. doi: 10.1101/gr.240705.118 (PMC6499312; doi:10.1101/gr.240705.118)
Supplement: Supplemental Material [file supp_gr.240705.118_Supplemental_file_1.zip › contigs/annotated_contigs/DB78/contig.2.DB78_length_579_mean_cov_9.26424870466.docx]

**DB78_length_579_mean_cov_9.26424870466**

```
GTTTCTGTATCACTTTTTATCTAACTTTTACTTTTTTTCTTTCATAGCATGAATCACTATCTGGCATAGGATATATTTGTTTAATGTAT
 >chr1:72140494-72140807 + E=1e-173 p=2e-02
GTTTTCATCCAACAGAATGTAGGCACCTTGAAACCTGCCTGTTTTGTATTCTTAGCAACCTTACCTAACACTTGTAACATTCCAACACT

GAATAAAGAAAAACTGAATATATACCAGATGTTGTATACATTGCATAAATATATATGTGATACATACGTATGTGATCTATATATGTGAT

CTATATATGCAATATATAAAATGCATGTTTATTCATATATATA|TAT|TTTAATTTATGTTAAATTGTCAAAGCCTAAATTAATGAATA
                                            >chr1:72143071-72143340 - E=3e-150
GCAAATGTGAACCTCCAAATGGGCTCATGTGGAAATATTCCTATTTGTGGTTATCTTTTTAAATTATATTAAGGGTTTGCTCTTTTGTG

GCTGTAAAATAAATATGATAACTTTACACAAGAACCCAATGTTCACTCAGTGAGTGTAGCAGAAATCAAATAAAAGCAGCCATGCAACT

CAAATTAAAGTTCAATATCATTAATTTAATTATAGTACATAATCATT
```
